# Conceptual understanding of social capital in a First Nations community: a social determinant of oral health in children

**DOI:** 10.3402/ijch.v74.25417

**Published:** 2015-01-22

**Authors:** Mohammad H. Salehyar, Louanne Keenan, Steven Patterson, Maryam Amin

**Affiliations:** 1 School of Dentistry, Faculty of Medicine & Dentistry, University of Alberta, Edmonton, Alberta, Canada; 2 Department of Family Medicine, Faculty of Medicine & Dentistry, University of Alberta, Edmonton, Alberta, Canada

**Keywords:** social cohesion, dental health, Aboriginal, native, indigenous, qualitative study

## Abstract

**Objectives:**

The purpose of the study was: (a) to better understand the concept of social capital and its potential role in oral health of children in a First Nations community and (b) to identify the strengths and resources in terms of social capital and a health promotion model that the community has at its disposal to address its oral health issues.

**Methods:**

In this qualitative case study, participants were purposively selected in a First Nations community: Seven individual interviews and two focus groups involving 18 parents/care givers were selected. Putnam's concept of social capital guided all the interviews. The interviews were recorded and transcribed verbatim. Thematic analysis was employed using the NVivo software.

**Results:**

The community was close-knit and seemed to have strong moral fibre, which encouraged members to help each other. A strong bonding social capital was also found among the members, especially inside the clans (families). A need for improvement in bridging social capital that would help the community to reach external resources was observed. While members of the community were actively involved in religious rituals and cultural ceremonies, more efforts seemed to be required to recruit volunteers for other events or programs. Active engagement of community members in any program requires that members be given a voice as well as some ownership of the process. Mobilizing or building community's social capital can play a role when planning future interventions.

**Conclusions:**

A better understanding of social capital may enhance the community's investment and efforts by reinforcing healthy oral behaviours and improving access to external resources. With more dynamic collaboration, it may be possible to create more sustainable community-based oral health promotion programs.

The prevalence of early childhood caries (ECC) in Canadian Aboriginal populations has been shown to range from 25 to 72% (1). Aboriginal children aged 3–5 have 5 times more dental decay compared to age-matched non-Aboriginal Canadian children (2). Because young children with ECC often present with behaviour management challenges, they commonly receive oral rehabilitation under general anaesthesia (GA) (3). Repeated dental surgeries to treat new carious lesions are also quite frequent among these children (4). The shocking numbers of children who continue to suffer from extensive tooth decay, and the financial burden of this situation to the provincial and federal governments, make the oral health of Aboriginal children in Canada a priority that demands in-depth investigation.

In recent years, a paradigm shift has occurred for predicting oral health outcomes by combining psychosocial and environmental factors with traditional biological measures (5). Current health promotion models suggest a multilevel approach targeted at the individual, family and community levels. One such conceptual framework for children's oral health classifies determinants in this manner and therefore expands the traditional focus on individual (and possibly family) determinants to also include community factors (5). In this model, the family and individual levels are situated within the community level, indicating that the community factors have a broader and more dominant role in shaping children's oral health.

Among community-level factors, there is growing evidence in public health literature supporting the health implications of social capital as a social determinant of health (6–9). The concept of social capital has generated ongoing debate in multidisciplinary health research literature during the past 2 decades. Among the 3 scholars providing initial theoretical contributions (Bourdieu, Coleman and Putnam), Putnam's work has received the most attention in the health literature within the extensive work of public health researchers (10). In Addition, Robert Putnam has received much of the credit for popularizing the concept within health research, by expanding upon the social science's theory, which has been viewed to offer only obscure and abstract conceptualization (11). Putman has defined the term as “features of social organization, such as trust, norms and networks that can improve the efficiency of society by facilitating coordinated actions” (12). Putnam initially equated social capital with networks of civic engagement, reciprocity, honesty and social trust, and thereafter added other consequences of networks, such as altruism, volunteering and philanthropy, as alternative indicators of social capital (10). As a health promotion strategy, community social capital can be mobilized. Mobilizing collective social capital is related to the “community development approach” of health promotion (13).

Communities with high versus low levels of social capital have better general health and lower levels of mortality, morbidity and violence (14–16). In terms of oral health, social capital has been shown to positively impact dental caries (17, 18), dental injuries in young populations (19) and the number of natural teeth in elderly people (20, 21). In contrast, informal social control, resulting from strong social capital, has been reported to cause stress and frustration in Japanese youth resulting in impaired oral health because of neglect in oral hygiene and improper eating habits (22). Yet, the role of social capital has not been fully explored in oral health (23) and, more specifically, little is known about the role of social capital in the oral health of preschool children in general and in First Nations children in particular.

The aims of this study were: (a) to better understand the concept of social capital and its potential role in oral health of children in a First Nations community and (b) to identify the strengths and resources in terms of social capital and a health promotion model that this First Nations community has available to address its oral health issues.

## Methods

### Study design

Ethics approval was obtained from the University of Alberta Research Ethics Board. Qualitative methods were thought appropriate for this study because there has been little research into the concept of social capital in oral health. It also has a great potential to illuminate some of the ongoing debates regarding the definition, utility, applicability and impact of social capital in relation to health (24).

A case study approach was selected because it is the preferred empirical research strategy for examining a contemporary phenomenon in depth and within its real-life context. The case study inquiry: (a) relies on multiple sources of evidence and (b) benefits from previously developed theoretical propositions that guide data collection and analysis (25). Putnam's theory of social capital (12) informed the study's design, data collection and data analysis.

### Setting and sampling

Through his previous experience working within this First Nations community in Alberta, the first author (MHS) – a non-Aboriginal graduate student – was approached by a community member who expressed the community's concerns about their children's oral health. The previous working relationship between the community and the first author (MHS) was crucial in building trust among the partners. A community research coordinator was collaborating with the study who had extensive experience in coordinating health research projects with First Nations and Métis communities, and reviewed the study design and instruments with the advisory committee to ensure their cultural appropriateness. Individual and focus group interviews were planned with a purposive sample of individuals who lived or assigned themselves as a member of the community. The intention was to achieve a sample representing those most concerned with and/or informed about the oral health of the community's children, as well as others who could provide insight into the structure and function of the community. Individuals meeting this inclusion criterion were informed about the study and invited to participate by telephone or letters mailed by the research coordinator, the health director and health workers in the community. Individual interviews were first undertaken with a small sample of community Elders and health workers involved in the oral health care of the community. Subsequently, additional interviews were conducted individually with other key informants – serving as health experts with the community – to explore some of the community health issues and to shed light on some of the applicable complexities related to the cultural background and history of the community. Finally, focus group interviews were conducted with a larger sample of mothers/caregivers who cared for preschool children within the community. The research team gained full, oral and audio-recorded informed consent prior to conducting any interviews.

### Data collection

The community advisory board provided input on the interview/facilitator guide that was used for the individual interviews and focus group sessions with community members. The concept of social capital was introduced to the participants using the terms “community social assets” and “community strengths,” and was stated to involve participation in community activities, volunteering, mutual trust, care and help of others, and neighbour and family connections. Open-ended questions were posed, relating to the social and environmental conditions of their community, social capital and how the social capital of the community could be used for the success of future oral health interventions (Appendix 1). Probing questions and comments were reflected back to encourage the participants to fully describe their thoughts, worries or concerns. The researcher deliberately wove in key points that identified from previous interviews, to allow for the refinement of existing categories or new codes. Both the research coordinator and the first author (MHS) facilitated all interviews. Each individual interview and focus group discussion lasted for approximately 60 to 90 minutes. The interviews were conducted in a community health office and the focus groups in community gathering places. Data collection and analysis were simultaneous and interviews were continued until data saturation was obtained.

### Data analysis

All the interviews were transcribed verbatim by a professional transcriber, and then reviewed and cross-matched with the recorded interviews by the first author (MHS). The transcribed texts were exported into NVivo 9 software for analysis.

The undertaken thematic analytical approach was deductive or concept-driven in order to allow interpretation of the findings. The categories which the codes represented came from Putnam's social capital and Ottawa's charter of health promotion concepts plus reading through the transcripts and other documents. A collection of codes were constructed as a list of key thematic ideas, at first, and were used during coding of the data (26). However, we changed the list of codes during analysis as new ideas and new ways of categorizing are detected in the text.

The thematic analysis of the data involved careful reading and re-reading of the text to get familiarized with it and to decide what it was all about. We undertook “intensive reading” when coding and paid close attention to all the details and constantly asked some basic questions that helped us to get started: What is going on? What are people doing? What is the person saying? How do structure and context serve to support, maintain, impede or change these actions and statements? (26)

Then the codes were combined into themes that describe the data. We looked at how the themes support the data and the overarching theoretical perspective. If the analysis seemed incomplete, we went back and found what was missing. A comprehensive analysis of what the themes contributed to understanding the data were done. We tried to define what each theme was, which aspects of data were being captured and what was interesting about the themes.

When we wrote the report, we made decisions on which themes made meaningful contributions to understanding what was going on within the community. To prepare for reporting the findings, exemplars for each code and category were identified from the data (27). Trustworthiness was achieved through reviewing findings with colleagues and maintaining a careful audit trail (28). We also conducted “member checking.” This was where we went back to the community to see if our description was an accurate representation. During this community gathering, feedback was sought to verify the interpretation of the data (member check) (28).

## Findings

Two Elders (one of each gender) and three health workers were interviewed in the first stage. Two additional interviews were completed with experts who had a long history of working with the community. Two focus groups were then conducted with 18 mothers/caregivers (4 males and 14 females, aged 21–53, all having First Nations Treaty status). The name of the community and the participants is not revealed due to the historical stigmatization of the First Nations people. Verbatim quotes from participants are indicated in quotation marks. To respect the privacy of the participants and maintain confidentiality, no real names or initials were used.

### Description of the community

As the community and its characteristics are central to the concept of social capital (29), we asked a series of open-ended questions about the social and environmental conditions of the community itself from our participants in addition to the questions regarding social capital. When asked to describe the community, participants talked about the “close-knit nature” as one of their community strengths. They believed the community members were “all connected” because they knew each other very well and the relationships were very close and, as a result, strangers could be easily recognized. It seemed that the community was built up around different clans or families that historically had conflicts with each other. But despite this, they believed “the spirit of the community” keeps them all together and inspires them to support each other when needed. Participants also talked about a strong religious belief among the community members and said that they were trying to find a harmony between their religious beliefs and their traditional and cultural background as First Nations people.

**Fig. 1:**
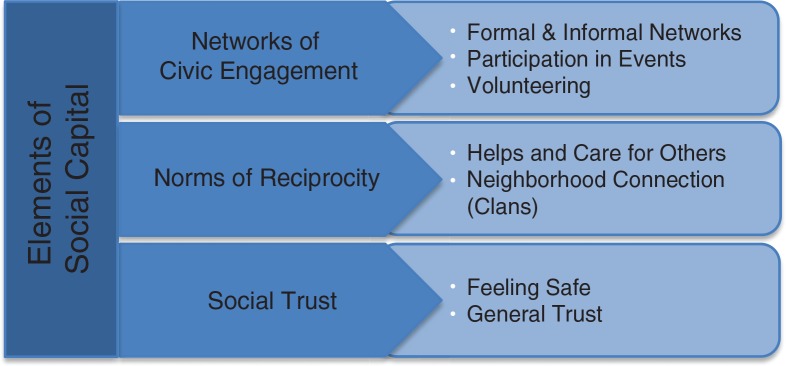
Elements of social capital according to Putnam and identified categories.

Two main theoretical themes were used to interpret the findings: social capital and oral health promotion. The findings related to Putman's theory of social capital were identified within 3 categories (Fig. 1). The participants’ citations regarding each category were demonstrated in Tables I–III.

**Table I:** Elements of social capital according to Putnam (networks of civil engagement) and identified categories

Elements of social capital	Networks of civic engagement	Formal networks	Elders’ Association: “The elders want to give back to the kids and want to educate children about cultural teachings, cultural preservation, to make sure that the language and the tradition isn't lost.” Associations and groups which get their financial support from the governmental programs or several charities: “Well, we do have organizations, they're not a registered societies or anything … but we do have women's group, and we do have ball recreation associations where all our children, like different types of recreation, are involved in, skiing, skating ….”
		Informal networks	“… when I run Alcoholic Anonymous (AA), I just run it from my own pocket, food is the way, so I just buy food and I run it and just people come that need help, that go to AA. I know there's a Narcotics Anonymous, I've never been to that association. I don't know if they have government support of any kind and I don't get government help. I don't get anybody's help.”
		Participation in events	“The spiritual and cultural gatherings are always packed with people … in the memorial dance people come and help, they like the interaction, they like the visitors, they like serving.” “… but in the healing community events … mostly staff, the odd community member came. I think one of the days they had like 10 people but it's a big community ….” “I think it's lack of communication. When things come up like this and when they say an events gonna happen, they don't share to anybody until it, last minute. Maybe if they notify about [it] a month early, notify the people what's gonna happen, maybe then, you'll have more people interested in helping out.”
		Volunteering	“nobody is interested in volunteering and donating their time” “… it could be easy and it could be difficult; depends on the interest of the community members.” “… for hockey and baseball, you would find, if there was something geared towards sports and they needed that help, of course people that roll in that circle are going to support it right, so they're going to volunteer to make it happen and then you have your cultural ceremonies, those people that wanna see that happen will move in that direction too, would give their support.” “There is some volunteers, those that are dedicated, those that do care but not always though. I think a lot of volunteer also happens during ceremonies, our sun dances, and our sweat lodges. People get together, a lot of men get together to set up, the sun dance lodge, nobody gets paid to do that, they all come together to do that. The women will cook, nobody pays them to do that but if I ask them to go and supervise the kids at the camp, how much (laughing) how much you gonna, or security work, yeah, things like that they will not volunteer.”

**Table II:** Elements of social capital according to Putnam (norms of reciprocity) and identified categories

Elements of social capital	Norms of reciprocity	Helps and cares for others	“A lot of people here are basically caring people; community members all have something they can give to the community that benefits all of them.” “I like the fact that we do try to help each other and a lot of people do make the effort and we're a small community and people still keep trying. That's a good thing.” “If I need ten dollars or something, I'll never ask. That's just the way my mom had raised us.”
		Neighbourhood connection (clans)	“… I could go ask for help if I needed it … like most of the time what you find is that people are more than willing to help if you need it that way, if for instance I needed you to give me a tug out of the ditch, [be]cause I went in the ditch, if somebody goes by, they'll say oh can I help you or can I take you somewhere or something like that … they won't just leave ya stranded that way if they know you're in trouble ….” “people here are kind hearted to each other and when they're not under the influence [of alcohol or drug] they really care about each other.”

**Table III:** Elements of social capital according to Putnam (social trust) and identified categories

Elements of social capital	Social trust	Feeling safe	“When I was a kid, we never used to lock our doors, but now you can't even do that [be] cause there's too many people that have no respect for another person's stuff, property and that. They don't have any respect for someone else, that means they don't have respect for themselves and they just go in someone else's home and take things that don't belong to you ….”
			“… with all the drugs and alcohol and prescription drugs, I don't know if it's as safe as it used to be.”
			“… and again that leads to alcohol and drugs and young people who are hooked on that stuff they're gonna do whatever, whatever means they can do to get, to get whatever it is they want ….”
		General trust	“… it is a small community, people know each other and know who to trust and who not to.”
			“… Well, when I say clan systems fighting amongst one another or historical grudges. I mean sometimes, someone can bring up something that's a hundred years old and still carry that grudge and not trust another, a different clan or even different family members or something to that respect. And a lot of times that do come up, there are disputes, clans’ disputes ….”
			“… but in terms of trusting people with my kids, no. I don't trust anybody, other than my husband and I got other family, adopted kids that I trust with my kids, but other, other than, between my husband and myself, I have a very hard time trusting others because of the sexual abuse, I was sexually abused here as a kid, I grew up like that, and I know it's still happening, I know it's still in the community and I know that's what keeping people down is that what they suffered as children, they're hiding it inside and that's the alcohol drug abuse ….”
			… there is a big gap in the [community]between the rich, what who think they're rich and who is poor, who they consider to be poor. So, those gaps kinda interfere with progress … It's a social gap.
			“… different family living together inside the community and these families are clans or classes that are living inside [the reserve]. The bigger the family is, they have a better chance to run for elected position and when they got to that point, they do whatever favour their family. There are exceptions where good people are elected in make good decisions, so you can't just blanket everybody but the controversy is because of that scenario.”
			“… generally, it is the largest family groups, the largest clans that have the power to put into council the one's that they want with the hope that they are going to be favoured and received the houses. The houses and the jobs and the benefits that come from having chief and council on your side. What happens then is that the families that don't have representatives on council are afraid they don't get the jobs, they don't get the houses and that fear is in a lot of, to a large extent realistic and that starts the wars. There is jealousy about who gets the housing, who gets the jobs, who gets the benefits of educational grants, so that's what that rivalry is about.”

#### Networks of civic engagement

Social networks, Putnam's first element of social capital, can be classified as formal or informal. Participants talked about different formal networks established by political, educational, religious and recreational organizations/associations. Examples included the government-funded justice committee that assists people with legal issues or when navigating the justice system and the Elders’ Association that helps community youth understand and connect with their First Nations tradition and cultural background. Some of these associations or groups receive financial support from government programs or charities. Informal networks also seemed to play a major role in this community. While a strong connection existed among the members in each clan, little interaction seemed to occur among different clans. A good relationship was reported amongst residents in each neighbourhood because the neighbours were mostly members of the same family. This could be a reflection of social ties, or bonding social capital, in this community. On the contrary, participants talked about rivalry among the clans, including “political setbacks, and historical clan disputes.” Nonetheless, they believed it is the spirit of the community that keeps them together, since “They might fight amongst each other, but when a need is there everybody comes together.” Another participant emphasized, “… there is situations where people are divided, but in the end, we're a community. A very close community ….” Participants also mentioned other informal support groups to help residents with their day-to-day life in the reserve, such as the Alcoholics Anonymous (AA) support groups.

The extent to which people participate in social and civil activities is another important component for measuring social capital in a community. In the community, people seem to participate well in events that are rooted in the community. Elections, spiritual and cultural gatherings, and sports were listed as the most well attended events, attracting many volunteers offering various supports. However, events and programs originated and implemented by “outsiders” usually fail to garner support from community members who do not have a strong sense of ownership of the program. In terms of health promotion programs, this finding suggests poor program participation, momentum and outcomes. In addition to this lack of sense of ownership, improper communication was another reason for low engagement of the members in community events.

A mixed response about volunteering was received from the participants. While some believed it was difficult to find volunteers unless an incentive is offered, because “nobody is interested in volunteering and donating their time,” others were more optimistic. Overall, it seemed that for events initiated by the community, finding volunteers was not problematic; conversely, those imposing events/programs on the community would had difficulties with volunteerism. Volunteer engagement may be a result of one's feeling connected with the activity, whereby the individual sees the benefit that their contributions made to the overall success of the program (Table I).

#### Norms of reciprocity

The participants talked about this element in terms of “help and care.” For some circumstances, because the community is close knit, members are willing to provide help to each other. One participant reflected that, “A lot of people here are basically caring people; community members all have something they can give to the community that benefits all of them.” However, some hesitation in asking for help was observed among the band members because they believed that they should “never go begging for anything” and should take care of their needs. Nevertheless, most community members seemed confident that when they need help, they would receive it without any delay. One participant described the community members as “just inbred caring people that are kind hearted” (Table II).

#### Social trust

Participants perceived the subject of trust as feeling safe and a general trust inside the community. While some participants thought that, overall, the band is “a safe community,” others described the “old days” to be much safer. Addiction and alcohol abuse were mentioned as reasons for the current lack of safety in the community: “… with all the drugs and alcohol and prescription drugs, I don't know if it's as safe as it used to be.” One of the focus group participants described sexual abuse as an example of a breach of trust in their community. It also appeared that people have more trust in the members of their own clans than in other community members or strangers. The rivalry or historical disputes among different clans appear to make trusting others more difficult. Moreover, the intergenerational impact of residential schools (IGIRS) was described as the cause for addiction, substance abuse and sexual abuse, and as a major barrier for social trust.

Social inequalities and the existence of different social classes seemed to be another reason for low levels of social trust in the band. One participant felt they were being treated as second-class community members because of getting married to a family in “the lowest strata of the community.” Another participant explained: “What happens is that the families that don't have representatives on council are afraid they don't get the jobs, the houses and that fear is to a large extent realistic and that starts the wars. There is jealousy, so that's what that rivalry is about” (Table III).

### Oral health promotion

The second major theme, used for interpretation of the qualitative data, was oral health promotion defined by Ottawa's charter. Needs of the community and resources regarding oral health of the community as a whole and of their children in particular were brought up by the participants. It appeared that pre-schoolers had never received an oral screening examination and, as a result, their oral health status was unknown. The available information related to children's oral health status seemed to be mostly based on anecdotal claims. One of the Elders expressed a need for professional help to gather necessary baseline information about their children's oral health status, “I suppose, if we knew where we're at, we could lobby. If we knew exactly where our babies and our kids are at in terms of dental help ….” Participants also stated that the community members have used all their available local resources to address the oral health issues of their children but that the problems have not been resolved. When asked, “What can we [the community with the help from the research team] do?” to address the oral health of children, an obvious frustration was expressed: “That's where we bang [our] heads now.” This community has a history of constructing supportive environments through various health projects by creating formal and informal networks in the community. For instance, Elders took the initiative to create an association (formal network) dedicated to helping the youth: “… the elders wanna give back to the kids and educate our children about cultural teachings, cultural preservation, to make sure that our language and our tradition isn't lost. This is a very unique asset for our community and our children.”

Other examples of community actions to address their health concerns were the establishment of an informal network such as AA as a volunteer support group, collecting base line data for suicide or sexually transmitted diseases (STD) prevention, and the organization of a rally on awareness about diabetes.

We asked about the potential benefits of their previous experiences towards developing future collective community actions for addressing the oral health issues. In response, one of the participants explained: “Coming in with programs and giving money to do things is not the answer. The answer is to empower the people to do things by themselves for themselves.”

While participants seemed to be confident about their ability to act and make changes towards a better condition, they also expressed their need for external support and resources. One of the individuals who worked closely with the community suggested:

You [the research team] just have to get people excited about the chance of improving [their current situation]. You have to have enough Elders and clan leaders on side to promote it within their own families, … so people will make the right decision with enough information.

Participants indicated that it is possible to plan for addressing the oral health issues, but acting on those decisions requires financial support. One Elder also explained that the community alone was unable to address the issue of oral health and required some form of external support: “Well, I think if we had the ability then it would be done right. I really don't think we have the resources to make sure that this is met.” The only source of funding for health services inside the community is federal funding through First Nations and Inuit Health Branch (FNIHB) of Health Canada, and any changes in the funding would likely result in a loss of funding towards other programs – a scenario that this community cannot afford.

## Discussion

Social capital can differ within and between the communities. The level of social capital inside each community may be affected by the physical, environmental and economic characteristics of a community (29). Our community seemed to be a traditional society in which social relationships are based on personal bonds of friendship and kinship that are largely ascribed rather than achieved. As a result, individuals are committed to their own group and feel a sense of togetherness, but they also have very limited privacy (30). As Portes (31) noted, poor communities frequently depend on close interactions among kin and friends for their everyday activities to maintain survival. This characteristic, whereby neighbours help each other get along with each other and share values, may also be defined as social cohesion (10), which is used within the health literature interchangeably with social capital.

The “close-knit nature” of the community, described by the participants as one of their strengths, is a reflection of the bonding social capital that may lead to enhancing community solidarity. However, such a strong bonding may create a backlash that encourages unhealthy behaviours and resistance to change. If the community decides to implement a change, the members are expected to distribute the information widely and support each other in the process. However, if the network feels disconnected from the process, a strong bond among members may cause internal resistance and more stress for some members who are in favour of the change. Thus, the strong solidarity during change may amplify or minimize stress depending on the situation. In this community, the Elders association has played a major role in facilitating changes in this community. In a Northern Sweden study, community members were able to build an association-driven health centre through collective actions and “symbolized by the fact that nearly the entire community stands together behind a project” (32).

Although the community clans have strong level of bonding social capital inside their own networks which is described as thick trust (33), their bridging social capital seems to require improvement. Ongoing rivalry and historical clashes inside this small community seemed to contribute to a difficulty in building trust in others, which has been called as thin trust (33). Equality can be conceptualized in 2 dimensions: economic and equality of opportunity (34). Oversight of either dimension could affect the social trust and, consequently, the social capital in a community. In our study, participants pointed to inequity of opportunity described as over-representation of some clans, which would provide them with a stronger voice and more leadership positions and thus greater power. Our participants repeatedly linked the power and social class to unequal access to financial and educational opportunities, which together widen the gap between the different clans. One's lifestyle was seen to benefit greatly by power, which continually strengthened through re-election to the leadership. In fact, one of our participants mentioned that people within smaller clans are treated as second-class community members. As Patrick et al. (35) described, individuals with more favourable characteristics (in this case, power) are classified into a higher social status in the hierarchy, which subsequently determines the provision of rewards. This social hierarchy provides differential benefits to individuals who occupy different positions. As a result, classification acts as a process that formalizes inequality in the form of unequal access to valuable resources, such as quality housing, education and health care.

Putnam's concept of social capital is criticized for not addressing “gender” and “power” issues (36). People with more resources are more often invited into powerful networks and dominant societal groups that have more power to decide what networks are valuable and to include or exclude people from these networks (11). In retrospect, using Putman's framework, which considers equality a pre-condition for building trust among community members, was not entirely suitable for explaining the phenomena of power differences in our community. Since Bourdieu argued that unequal distribution and accumulation of capital (economic, cultural and social) is an explanation for the production and reproduction of social structure (10), it seems Bourdieu's concept (37) appears to be more promising for future work in First Nations communities because it provides explanatory insights into the power dynamics of these communities. Nonetheless, adopting Bourdieu's theoretical framework in a similar context requires an in-depth consideration of change in the analytical unit from the community to the individual level.

### Community development to foster oral health promotion

From the voices of the participants, it appears that active engagement of community members in any program requires that members be given a voice as well as some ownership of the process. This finding aligns with the conceptualization of health promotion as discussed in detail by Robertson and Minkler (38). Professional consultants or research teams should plan to work with the community to empower people to take responsibility for the upcoming changes. In addition, any community development effort should complement rather than replace the additional systematic changes required to promote the oral health of this population as reiterated in the First Nations Oral Health Survey (FNOHS) report (39):

It must be noted that despite prevention efforts, change must also occur on a much larger social scale. Disparity with respect to broad health determinants (education, poverty, over-crowding, substance use, and provision of care) is highly linked with poor oral health. Smaller-scale prevention efforts are only so effective without the recognition and improvement on these societal-level disparities.

This recommendation for promoting community development interventions is particularly suitable for the community that has some previous experience in this realm for addressing other health issues such as prevention of suicide and substance abuse.

With collaboration between research team and community partners, it may be possible to lobby and bring about new changes. However, the community faces significant difficulties with limited funding and the current number of healthcare personnel. Therefore, expecting any of these individuals to work voluntarily in a new intervention, that is, oral health program is not a feasible expectation. As a result, this community is greatly restricted in their ability to initiate or to implement any oral health program that would benefit the children without any additional external human and/or financial resources.

## Conclusion

This exploratory pilot study employed a novel approach of investigating the concept of social capital in relation to the oral health of First Nations people and its potential role in improving children's oral health. The community was “close knit” with strong intra-family bonds and a high commitment to contribute to community activities. This strong bonding social capital could serve as strength to the community to achieve changes should the necessary resources become available. Change may be difficult to realize if their efforts are thwarted by failure to strengthen their bridging social capital; they need to overcome class/clan and power inequalities in order to facilitate access to required external resources. In addition, with a more dynamic collaboration between experts and community partners, it may be possible to lobby for changes to create a more sustainable community-based oral health promotion program. The findings of this inquiry can be used to design additional research to identify in more depth the context of social capital for the First Nations communities and/or individuals as a rural setting. In addition, the power and gender issues were exposed only to the extent that they justify more focused investigation.

## Appendix 1: Interview Guide

The moderator will start the session by introducing herself/himself to the participant along with a brief summary of the study. Then they will outline the ground rules that will guide the individual interview. The consent form will be distributed and participant will fill it out before starting of the discussion.

**Community**

**- How would you define or describe your community?**

**- How do you feel about the place you live in?**

**- Probing Questions:**

What is good about your community? What do you like about your community? What don't you like (if any)?

What makes your community different or recognizable from other First Nations communities?

How separate are poor and riches in your community? Is there a wide gap between the poor and the rich in your community?

**Social Capital**

**A. Participation in the Local Community**

**- Tell me about the participation of people in your community events, volunteering and support groups**.

**- Probing Questions:**

How often do you participate as a volunteer in the community events?

What activities do exist in your community that need participation of volunteers and local people?

Is it difficult to find volunteers for an event in the community? When was the last time that you participated in an event as a volunteer?

**B. Feelings of Trust and Safety**

**- Tell me about trust among people and feeling of safety in your community**

**- Probing questions:**

How safe do you feel your community is? Do you lock the door of your house after sunset? Do you feel safe to walk down the street after dark?

How much trust do you have in other people who live in your community? Are they trustworthy?

**C. Neighbourhood Connections**

**- How would you describe relation between people with each other and their neighbours?**

**- Probing Questions:**

How good is the relationship between you and your neighbours?

How comfortable are you to ask them for help when you need it?

Would you help your neighbours when they need it? Are they comfortable enough to ask your help?

**D. Family and Friends Connections**

**- Tell me about the interaction among families and friends**.

How much connection do you have with your family and friends?

How big is your circle of family and friends?

**Oral Health Promotion**

**1. Creating supportive environments**

What can the community (collective actions) do about oral health issues such as tooth decay? Is there any power/will or interest in your community to address this issue?

How can a supportive environment be created to address this issue?

Is it a priority for the community to address the oral health of young children?

**2. Strengthening community action**

What are the facilitators and barriers?

What internal and external resources do the community have that would help resolve this problem?

Do you know about COHI? Do you know about its services? How the community can improve the rate of using of the services of this initiative? What works and what doesn't?
